# Antimicrobial Activity of Few Medicinal Plants against Clinically Isolated Human Cariogenic Pathogens—An *In Vitro* Study

**DOI:** 10.5402/2011/541421

**Published:** 2011-06-08

**Authors:** H. Shyla Jebashree, S. Jayasurya Kingsley, Emmanuel S. Sathish, D. Devapriya

**Affiliations:** ^1^Department of Plant Biology and Biotechnology, School of Life Sciences, Loyola College, Chennai 600 034, India; ^2^Department of Conservative Dentistry and Endodontics, R.M.D.C.H., Annamalai University, Annamalai Nagar, Chidambaram, Pin code-608002, India

## Abstract

Hexane, ethyl acetate, ethanol and methanol extracts of *Psidium guajava, Terminalia chebula, Mimusops elengi and Achyranthes aspera* were tested against the dental caries causing bacteria *Streptococcus mutans* and fungus *Candida albicans* isolated from caries infected patients. All the four extracts of *P. guajava* showed activity against both *S. mutans* and *C. albicans*. Maximum zone of inhibition was observed in ethyl acetate of *P. guajava*. The four extracts of *T. chebula* and *M. elengi* showed antibacterial activity against *S. mutans*. *M. elengi* extracts and ethanol extract of *T. chebula* did not show any antifungal activity against *C. albicans*. Except for the hexane extract of *A. aspera*, the other three extracts showed activity against the tested microbes. The ethyl acetate *P. guajava* leaf extract showed the minimum inhibitory concentration (MIC) against *S. mutans* to be <0.076 mg/mL in both MHB and BHI. The *P. guajava* ethyl acetate extract was subjected to GC-MS.

## 1. Introduction

Dental caries is an infectious microbial disease that results in localized dissolution and destruction of the calcified tissues of the teeth [1]. *Streptococcus mutans *is known as the causative bacteria in the formation of dental plaque and dental caries. The acid producing *S. mutans* inhabiting the mouth causes damage by dissolving tooth structures in the presence of fermentable carbohydrates such as sucrose, fructose, and glucose [2]. The food debris, acid, bacteria, and saliva combine in the mouth to form a sticky substance called “plaque” that adheres to the teeth. If plaque is not removed thoroughly and routinely, tooth decay will not only begin but flourish [3]. 

 Persistent dental disease is painful, and most importantly, it has also been suggestively linked to diabetes, high blood pressure, heart disease, and multiple sclerosis later in life. The pain can be worsened by heat, cold, or sweet foods and drinks [4, 5]. Treatment often prevents further infection of the tooth structure. Early treatment is less painful than treatment of extensive decay. Dental caries can also cause bad breath and foul tastes. In highly progressed cases, infection can spread from the tooth to surrounding soft tissues which may lead to an edentulous mouth [6].

 Antibiotics such as penicillin and erythromycin have been reported to effectively prevent dental caries in animals and humans, but they are never used clinically because of many adverse [7]. Recent natural remedies with the use of medicinal plants, which are good reservoirs of chemo-therapeutants can be, contributed as an alternative for antibiotic effects such as hypersensitivity reaction, supra infections, and teeth stainings.

It has been well documented that medicinal plants confer antimicrobial activity towards oral bacteria [8, 9]. The literature survey of the folklore medicine reveals the use of *Psidium guajava* leaves to maintain oral hygiene, dried fruit of *Terminalia chebula* as an anticaries agent; stem of *Achyranthes aspera* for the treatment of tooth—ache and stem of *Mimusops elengi*, strengthens the gums [10–13]. Despite several anticaries agents being available commercially, the search for an effective agent still continues. Natural products have shown to be a good alternative to synthetic chemical substances for caries prevention [14].

Though recent reports show the antibacterial activity of* P. guajava *and *T. chebula* against the cariogenic bacteria, its antibacterial and antifungal activity with different solvent extracts are screened in this study. Knowing the fact that little literature is available on the anticariogenic property of* A. aspera and M. elangai, *the study is focused on assessing the plant extracts with different solvents. Hence, for the present investigation,* S. mutans*,* C. albicans *are the bacterial and fungal strains selected as target organisms from infected patients and screened using hexane, ethyl acetate, ethanol and methanol extracts of leaves of* P. guajava, *dried fruit of *T. chebula, *roots of *A. aspera*, and sticks of *M. elengi. *Once the antimicrobial property of the plant extracts is screened under *in vitro* condition against oral pathogens, *in vivo* trials can be carried out for the treatment of dental caries by external application on the caries tooth or as a preventive mouth rinse.

## 2. Materials and Methods

### 2.1. Microorganisms

The human dental caries pathogens, *Streptococcus mutans *and fungal pathogen *Candida albicans *used in this study, were isolated from caries infected patients of the Department of Endodontics and Conservative Dentistry in association with the Department of Microbiology, Meenakshi Ammal Dental College, Chennai.

### 2.2. Media Used

Thioglycolate broth (TGB) and brain heart infusion broth (BHI) are the transport media used to maintain clinical dental caries sample in viable condition. Thioglycolate broth (TGB) contained per liter of deionised water: 15 g casein enzyme hydrolysate, 5 g yeast extract, 5.5 g dextrose, 2.5 g sodium chloride, L-cystine 0.5 g, 0.5 g sodium thioglycolate with a pH of 7.1 at 25°C. Brain heart infusion broth (BHI) contained, per liter of deionised water: 200 g calf brain infusion from, 250 g brain heart infusion from, 10 g protease peptone, 2 g dextrose, 5 g sodium chloride, 2.5 g disodium phosphate with a pH of 7.4 at 25°C.

Growth media used in examining the samples at aerobic condition includes, nutrient agar (NA), blood agar (BA) and MacConkey agar (MAC). Nutrient agar (NA) contained, per liter of deionised water: 5 g Hi veg peptone, 1.5 g Hi veg extract, 1.5 g yeast extract, 5 g sodium chloride, agar 15 g with a pH of 7.4 at 25°C. Blood agar (BA) is prepared by adding 20 mL of sheep blood to 200 mL of nutrient agar media as prepared like the above-mentioned composition. MacConkey agar (MAC) contained, per liter of deionised water: 17 g peptic digest of animal tissue, 3 g Protease peptone, 10 g lactose, 1.5 g Bile salts, 5 g sodium chloride, 0.03 g neutral red and agar 15 g with a pH of 7.1 at 25°C. For the examination of pathogenic fungi from dental caries sample, Sabouraud's dextrose agar (SDA) contained per liter of deionised water: 40 g dextrose, 10 g peptone and 20 g agar with a pH of 5.7 before autoclaving.

 Growth media used to examine the samples at microaerophilic condition are brain heart infusion blood agar + 20% sucrose (BHIBA + 20% sucrose), thioglycolate agar (TGA) and trypticase yeast extract cystine sucrose bacitracin agar (TYCS20B) [15] (Schaeken et al. 1986), a medium for the selective isolation of* S. mutans* contained per liter of deionised water: 40 g trypticase soy agar (TSA), 5 gm Bacto agar (Difco), 10 g yeast extract, 200 g sucrose. The medium was sterilized and cooled to 55°C 200 IE bacitracin was incorporated. BHI broth with agar served as brain heart infusion agar used for culturing of* S. mutans* under microaerophilic condition. TGB with agar 2 gm/lt served as thioglycolate agar (TGA) used for culturing of* S. mutans* under microaerophilic condition. 

Growth media used to examine the samples at anaerobic condition is BHIBA + 20% sucrose above provided with H_2_ and N_2_ gas in the anaerobic jar. 

Mueller-Hinton agar (MHA) contained per liter of deionised water: meat infusion 2.0, casein hydrolysate 17.5, starch 1.5, agar-agar 13.0, Mueller-hinton broth (MHB), and brain heart infusion broth (BHI) are used for the antimicrobial susceptibility testing.

### 2.3. Collection and Recovery of Caries Sample

The samples from patients were collected with strict asepsis condition. Prior to the collection of dental caries sampling, patient was made to rinse the tooth with water, and it was isolated with a rubber dam. The tooth and the surrounding field were cleaned with 3% hydrogen peroxide and then decontaminated with a 2.5% sodium hypochlorite solution. The food debris on the chewing surface were removed using a dental excavating instrument. The dental caries sample was collected from the patient using an excavator under aseptic conditions by a clinician and was introduced into the 2 ml broth of TGB or BHI in appropriate sterile screw cap bottles. The clinical samples were mixed well using a magnetic stirrer before incubation. The samples were then inoculated using the streak plate technique on to the appropriate culture media under various culture conditions (four separate media on aerobic, three separate media on microaerophilic, and one media on anaerobic culture conditions for each patient sample) as shown in Table 1.

### 2.4. Identification of Dental Caries Pathogen

The detailed colony morphology and Biochemical characterization [16] (Holding and Colee, 1971) of the pathogens involved are shown in Table 2.

### 2.5. Plant Materials Collection

We selected four Indian medicinal plants for antimicrobial assay, based on their ethnomedicinal and traditional uses against infectious diseases based on literature survey and interaction with herbal healers. The plants were collected from Arignar Anna Medicinal Farm, Anna arch, Chennai. Roots of *Achyranthes aspera* (amaranthaceae), sticks of *Mimusops elengi *(Sapotaceae), leaves of *Psidium guajava *(Myrtaceae); and dried fruit of *Terminalia chebula *(Combretaceae) were collected and identified with the plant taxonomist Dr. M. Ayyanar, ERI, Loyola College, Chennai.

### 2.6. Preparation of Crude Extracts

The plant parts were shade-dried and powdered and used for extraction, 100 g of dry powder was taken in an aspirator bottle, 300 mL hexane (1 : 3 W/V) was used and the mixture was shaken occasionally for 48 hour. Then, the extract was filtered. This procedure was repeated three times and all extracts were decanted and combined. The extracts were filtered before drying using Whatman filter paper no. 2 on a Buchner funnel, and the solvent was removed by vacuum distillation in a rotary evaporator at 40°C for quantitative determination; the extracts were placed in preweighed flasks before drying. The remaining plant residue was extracted with ethyl acetate, ethanol, and methanol sequentially [17].

## 3. Antimicrobial Susceptibility Assay

### 3.1. Disc Diffusion Assay

Antimicrobial activity was carried out using disc-diffusion method [18]). Petri plates were prepared with 20 mL of sterile brain heart infusion agar (BHI) for *S. mutans* and Mueller-Hinton agar (MHA) (Hi-media, Mumbai) for* C. albicans*. The test cultures (100 *μ*L of suspension containing 10^8^ CFU/mL bacteria) were swabbed on the top of the solidified media and allowed to dry for 10 min. The tests were conducted at three different concentrations of the crude extract (200 mg crude extract dissolved in 5% dimethyl sulfoxide (DMSO), respectively, 5 mg and 2.5 mg per disc). The sterile 6 mm disc (Himedia) impregnated with different concentrations of extracts. The loaded discs were placed on the surface of the medium and left for 30 min at room temperature for compound diffusion. Negative control was prepared using respective solvent. Penicillin and Amphotericin-B (25 *μ*g/disc) were used as positive control. The plates were incubated for 24 h at 37°C. Zone of inhibition was recorded in millimeters and the experiment was repeated twice.

### 3.2. Minimum Inhibitory Concentration

The minimum inhibitory concentration (MIC) was performed according to the standard reference method [19]. The extracts were dissolved in water +2% dimethyl sulfoxide (DMSO). The initial concentration of extract was 5 mg/mL to 0.075 mg/mL. The initial test concentration was serially diluted two-fold. Each well was inoculated with 5 *μ*L of suspension containing 10^8^ CFU/mL of bacteria and fungi. The antibacterial agent penicillin and antifungal agent Amphotericin-B were included in the assays as positive controls. The plates with bacteria were incubated 24 h at 37°C. After incubation, 5 *μ*L of tested broth was placed on the sterile MHA and BHI plates and incubated at respective temperature. The MIC for bacteria was determined as the lowest concentration of the extracts inhibiting the visual growth of the test cultures on the agar plate. Three replications were maintained.

### 3.3. Gas Chromatography-Mass Spectrometry (GC-MS)

The active extracts were quantified using gas chromatograph (GC-MS-Shimadzu) equipped with a CPB-capillary column (mm inner diameter × 50 m length) mass spectrometer (ion source 200°C, RI 70 eV) programmed at 40°C–280°C with a rate of 4°C/min. Injector temperature was 280°C; carrier gas was He (20 psi).

## 4. Results and Discussion

Identification of the clinical isolates of *S. mutans* and* C*. *albicans* was done in collaboration with a microbiologist. Colony morphology, light microscopic properties and bio-chemical tests were taken into consideration in the identification of the isolated pathogens. *C*.* albicans *on SDA plates showed colonies that are rapidly growing soft, glistering or dull, smoother wrinkled, and white or yellowish cream in colour. The isolated bacterium showed alpha haemolytic *streptococci *producing a greenish discoloration with partial haemolysis around the colonies on blood agar (BA) plates. This is a characteristic feature for identification of the colony as* S. mutans*. Photomicrographs obtained from Gram's staining and the biochemical tests (Table 3) revealed that *S. mutans* is the bacterium recovered from the dental caries sample of the infected patients.* C*. *albicans *on SDA plates showed colonies that are rapidly growing soft, glistering or dull, smoother wrinkled, and white or yellowish cream in colour. The germ tube test confirmed the isolation of *C*. *albicans *recovered from caries infected patients. 

Four plants (*P. guajava*, *T. chebula, A. aspera*, and *M. elengi*) with four solvent extracts (hexane, ethyl acetate, ethanol, and methanol) were initially evaluated for their anticariogenic activity against* S*. *mutans* by the disk diffusion assay. All the four plants showed activity against *S. mutans. *Ethyl acetate extracts of the four plants showed high antibacterial activity against *S*. *mutans *than other solvent extracts. *P. guajava* ethyl acetate extract showed potential inhibitory action against *S*. *mutans* of 20 mm (5 mg/disc) and 18 mm (2.5 mg/disc).The results were summarized in Table 3.

The antifungal activity of the above-mentioned plant extracts was tested against *C. albicans*. Results revealed that all the *P. guajava* extracts showed activity against *C. albicans*, whereas all the *M. elengi* crude extracts was devoid of any activity against the isolated fungal pathogen. Except for the ethanol extract of *T. chebula, *three other solvent extracts showed activity against C. *albicans*. Ethyl acetate extracts of *P. guajava *and *T. chebula* showed significant activity against *C. albicans* 16 mm (5 mg/disc) and 14 mm (2.5 mg/disc). *A. aspera *extracts inhibited the growth of* C*. *albicans *but not hexane extract. The results are listed in Table 4.

Based on the preliminary screening assay, the *P. guajava* and* T. chebula* extracts were further evaluated to determine the minimum inhibitory concentration (MIC). MIC was determined as the lowest concentration of the extract, which inhibited the growth of the tested microorganisms. Results exhibit the profound and promising activity of *P. guajava* on BHI and MHB at <0.076 mg/mL against *S*. *mutans* with all the four extracts except for the ethanol extract on MHB showed MIC at 0.152 mg/mL. The antifungal activity against *C. albicans* of the hexane extract of* P. guajava *on BHI showed MIC at 0.152 mg/mL; 0.315 mg/mL for ethyl acetate and 0.625 mg/mL for ethanol and methanol extracts. On MHB, the fungus showed MIC at 0.315 mg/mL for *P. guajava *hexane and ethanol extracts, 0.152 mg/mL for ethyl acetate and 0.625 mg/mL for methanol extract. The results are tabulated in Table 5. 

The antibacterial activity of *T. chebula *against* S*. *mutans* exhibited MIC of <0.076 mg/mL for all the four extracts on BHI. MIC of the same extract on MHB showed <0.076 mg/mL for hexane, >5.0 mg/mL for ethyl acetate and ethanol, 0.625 mg/mL for methanol. *T. chebula* hexane extract for* C. albicans* showed MIC at <0.076 mg/mL on BHI and 0.625 mg/mL on MHB, ethyl acetate and ethanol extract showed MIC at >5.0 mg/mL both on BHI and MHB, methanol extract showed MIC at 0.315 mg/mL on BHI and 1.25 mg/mL on MHB, respectively. The MIC results are outlined in Table 5. 

The values of MIC reveals that the *P. guajava* extract against *S*. *mutans* on BHI showed no significant changes in value (<0.076 mg/mL) than MHB exhibiting anticariogenic activity.

 The ethyl acetate extract of *P. guajava* was further studied for its phytochemical constituents by subjecting to GC-MS analysis. The leaf extracts of* P. guajava *have some pharmacological activities, such as anti-inflammatory antidiarrhoeal besides antimicrobial activities [20, 21]. It has also been used extensively as a hypoglycaemic agent [22]. The cariogenic bacteria are known for its adherence ability to the tooth surfaces [23]. *P. guajava *extracts have been studied for their antiadherence effect on the cariogenic bacteria in the process of dental plaque formation [24, 25]. The active flavanoid compound gujaverin isolated from the methanol leaf extract of *P. guajava *has been demonstrated by Prabu et al. [26] to be a potential antiplaque agent by inhibiting the growth of *S*. *mutans*. However, in the present study, the ethyl acetate extract of *P. guajava* alone has shown proficient antibacterial activity with high zone of inhibition 20 mm (5 mg/disc) and 18 mm (2.5 mg/disc) although the MIC values are the same (<0.076 mg/mL) for both ethyl acetate and methanol extracts.

 Although *P. cattleyanum* has been reported to significantly inhibit the growth of *C. albicans P. guajava* is reported by us to be effective against the oral candidal infection with the potential activity of <0.076 mg/mL concentration [27]. An aqueous extract of *T. chebula* is evident to be an effective anticaries agent used as a mouth rinse [28]. Our results further elucidate the efficiency of *T. chebula *ethyl acetate extract to be an effective anticariogenic agent in inhibiting* S*. *mutans* and *C. albicans.*

 The study is a preliminary assessment of the easily available medications of plant origin which can be effective in the treatment of dental caries. To identify the active compound involved in anticariogenic activity of the very efficient *P. guajava* ethyl acetate extract revealed in the present study, the GC-MS results were obtained. Thirteen compounds were identified among which is caryophyllene a compound known for its curative efficiency as an anti-inflammatory and remarkable antibacterial activity [29–31]. The bioactive compound should further be evaluated for its anti cariogenic properties and should be subjected to *in vivo* trials before using as a preventive mouth rinse and components of tooth paste. This natural therapeutant can meet out the challenges faced in dental caries management and can be an efficient community-based healthcare system.

## Figures and Tables

**Table 1:** Isolation of the dental caries samples in various culture media under different growth conditions.

Culture method									
S. no.	Name of the organism	*Aerobic*				*Microaerophilic*			*Anaerobic*
		NA	BA	MAC	SDA	BHIBA +20% SUC	TGA	TYCSB	BHIBA +20% SUC
1	*Streptococcus mutans*	+	+	No Growth	−	+	+	+	−
2	*Candida albicans*	−	−		+	−	−	−	−

NA—nutrient agar; BA—blood agar; Mac—macConKey agar; SDA—sadouraud dextrose agar; TGA—thioglycolate agar; BHIBA + 20%—brain heart infusion blood agar + 20% sucrose; TYCSB—trypticase yeast extract cystine sucrose bacitracin Agar.

+: Present.

−: Absent.

**Table 2:** Morphological and biochemical identification for the confirmation of the isolated microbes.

S. no.	Name of the organism	Colony morphology	Grams rxn	Catalase	Oxidase	Sorbitol	Mannitol	Germ tube test
1	*Streptococcus mutans*	Hemolytic pinpoint colonies	Gram +ve cocci, scattered, and in small colonies	−ve	−ve	+ve	+ve	×
2	*Candida albicans*	White, large, round, opaque colonies or large creamy concave colonies.	Gram +ve budding yeast cells	×	×	×	×	+ve

+ve: positive; −ve: negative; ×: absent.

**Table 3:** Antibacterial activity of different solvent extracts of few plants against *Streptococcus mutans *isolated from cariogenic pathogens.

Name of the plants	Zone of Inhibition (mm)							
	Hexane		Ethyl acetate		Ethanol		Methanol	
	mg/disc		mg/disc		mg/disc		mg/disc	
	5	2.5	5	2.5	5	2.5	5	2.5
*P. guajava*	12	12	20	18	12	11	14	12
*T. chebula*	16	14	16	14	13	11	13	11
*A. aspera*	—	—	18	12	10	10	10	10
*M. elengi*	11	—	16	14	12	10	13	10

**Table 4:** Antifungal activity of different solvent extracts of few plants against *Candida albicans* from cariogenic pathogens.

Name of the plants	Zone of Inhibition (mm)							
	Hexane		Ethyl acetate		Ethanol		Methanol	
	mg/disc		mg/disc		mg/disc		mg/disc	
	5	2.5	5	2.5	5	2.5	5	2.5
*P. guajava*	16	14	16	14	15	13	15	13
*T. chebula*	12	11	16	14	—	—	11	10
*A. aspera*	—	—	15	14	12	14	13	12
*M. elengi*	—	—	—	—	—	—	—	—

**Table 5:** Minimum inhibitory concentration (MIC) of four solvent extracts of *Psidium guajava and Terminalia chebula* by microbroth dilution method.

Name of the plant	Isolated pathogen	Minimum inhibitory concentration mg/mL							
		Hexane		Ethyl acetate		Ethanol		Methanol	
		BHI	MHB	BHI	MHB	BHI	MHB	BHI	MHB
*P. guajava*	*S. mutans*	<0.076	<0.076	<0.076	<0.076	<0.076	<0.152	<0.076	<0.076
	*C. albicans*	0.152	0.315	0.315	0.152	0.625	0.315	0.625	0.625
*T. Chebula*	*S. mutans*	<0.076	<0.076	<0.076	>5.0	<0.076	>5.0	<0.076	0.625
	*C. albicans*	<0.076	0.625	>5.0	>5.0	>5.0	>5.0	0.315	1.25

MHB: Mueller Hinton Broth.

BHI: Brain Heart Infusion Broth.
